# Dietary patterns and recurrent pregnancy loss: a comparison of the American Heart Association diet, Mediterranean diet and others

**DOI:** 10.3389/fnut.2025.1565107

**Published:** 2025-06-04

**Authors:** Yan Ma, Qianqian Li, Rui Li, Liangjing Lu

**Affiliations:** Department of Rheumatology, Renji Hospital, Shanghai Jiao Tong University School of Medicine, Shanghai, China

**Keywords:** recurrent pregnancy loss, dietary patterns, adverse pregnancy outcomes, American Heart Association diet, Mediterranean diet, gestational diabetes

## Abstract

**Background:**

Recurrent pregnancy loss (RPL) presents a major challenge in reproductive medicine, with lifestyle factors, especially dietary patterns, potentially influencing pregnancy outcomes. This study aimed to explore the relationship between adherence to preconception dietary patterns and pregnancy outcomes in women with RPL.

**Methods:**

The study included 475 women with RPL at Renji Hospital, Shanghai Jiao Tong University School of Medicine. Participants completed a semi-quantitative food frequency questionnaire (FFQ) to assess adherence to six pre-defined dietary patterns at preconception: the American Heart Association Diet (AHA), Trichopoulou Mediterranean Diet (TMED), Panagiotakos Mediterranean Diet (PMED), Alternate Mediterranean Diet (AMED), Healthy Eating Index-2015 (HEI-2015), and Dietary Approaches to Stop Hypertension (DASH). Pregnancy loss, gestational diabetes mellitus (GDM), hypertensive disorders of pregnancy (HDP), and other adverse pregnancy outcomes (APO) (e.g., preterm birth, low birth weight) were ascertained using medical records.

**Results:**

Significant associations were observed between adherence to the AHA diet and reduced risks of pregnancy loss [adjusted RR (95% CI), highest quartile (Q4) vs. lowest quartile (Q1): 0.36 (0.17, 0.78), P-trend = 0.043], GDM [adjusted RR (95% CI), highest quartile (Q4) vs. lowest quartile (Q1): 0.28 (0.10, 0.75), P-trend = 0.006], HDP [adjusted RR (95% CI), highest quartile (Q4) vs. lowest quartile (Q1): 0.12 (0.03, 0.57), P-trend = 0.008], and other adverse pregnancy outcomes [adjusted RR (95% CI), highest quartile (Q4) vs. lowest quartile (Q1): 0.04 (0.01, 0.35), P-trend = 0.001]. Similar associations were found for the AHEI, AMED, and TMED diets regarding pregnancy loss, GDM, and HDP, while the PMED and DASH diets showed no significant associations. Additionally, higher levels of moderate-to-vigorous physical activity and lower energy and fat intake were associated with increased live birth rates.

**Conclusion:**

Greater adherence to the AHA diet during the preconception period was linked to lower risks of pregnancy loss, and adverse pregnancy outcomes. These findings support the AHA diet for patients with recurrent pregnancy loss, indicating that healthy dietary patterns may improve pregnancy outcomes and highlight the need for further research on their impact on fertility.

## Introduction

Recurrent pregnancy loss (RPL), defined as the occurrence of two or more consecutive miscarriages, affects a significant proportion of women globally and poses a substantial emotional, psychological, and physical burden (1, 2). While various factors have been identified as contributing to recurrent pregnancy loss, including genetic, immunological, hormonal, and anatomical causes, emerging evidence suggests that dietary patterns may also play a role in influencing pregnancy outcomes (3). Among the numerous dietary approaches, the American Heart Association (AHA) diet stands out due to its emphasis on heart-healthy eating habits, such as increasing the intake of fruits, vegetables, whole grains, lean proteins, and healthy fats, while limiting the consumption of processed foods, trans fats, and refined sugars (4). This diet has been widely validated for its benefits in reducing cardiovascular risk factors such as hypertension, dyslipidemia, and obesity, all of which are known to be associated with adverse pregnancy outcomes (5). Nutritional requirements during pregnancy are distinct from those of nonpregnant individuals, due to increased metabolic and physiological demands. Clinical guidelines recommend individualized dietary counseling based on maternal nutritional status and BMI to support optimal pregnancy outcomes (6).

Previous observational studies have highlighted the influence of specific nutrients and dietary patterns on pregnancy outcomes, suggesting that dietary modifications may play a significant role in improving fertility and reproductive success (7–10). In this context, a cohort study by Salas-Huetos and colleagues (11) found that adherence to the American Heart Association (AHA) dietary pattern prior to infertility treatment was associated with a reduced likelihood of pregnancy loss during treatment, further emphasizing the potential benefits of the AHA diet for reproductive health. However, despite these promising findings, there remains a gap in research comparing the effects of the AHA diet with other well-established dietary patterns, such as the Mediterranean diet, the Healthy Eating Index 2015 (HEI-2015), or the Dietary Approaches to Stop Hypertension (DASH), in women with recurrent pregnancy loss.

This observational study seeks to fill this gap by conducting a comparative analysis of the AHA diet and other commonly followed dietary patterns, assessing their respective roles in managing recurrent pregnancy loss (RPL) outcomes. By examining dietary patterns in women with a history of recurrent pregnancy loss, we aim to identify potential associations between specific dietary choices and improved pregnancy outcomes. Through this analysis, we aim to provide insights that could inform clinical recommendations and guide future research in the field of reproductive nutrition.

## Materials and methods

### Study population

This study was conducted at the Department of Rheumatology, Renji Hospital, Shanghai Jiao Tong University School of Medicine, from October 1, 2022, to October 1, 2024. The study included women aged 20–40 years with a history of recurrent pregnancy loss who were actively trying to conceive at the time of enrollment. Recurrent pregnancy loss defined as two or more consecutive miscarriages, intrauterine fetal death, or biochemical pregnancy loss and were actively trying to conceive. Exclusion criteria included a body mass index (BMI) outside the range of 18.5 to 30 kg/m^2^, the presence of major pre-existing chronic conditions, such as diabetes or hypertension, the presence of defined autoimmune diseases (e.g., Sjögren’s syndrome, rheumatoid arthritis, antiphospholipid antibody syndrome, or systemic lupus erythematosus), and women whose dietary habits had changed in the past year.

A total of 600 women were enrolled in the study and completed a baseline semi-quantitative food frequency questionnaire (FFQ) during their initial visit, which assessed their habitual diet and physical activity over the preceding year. In addition, preconception demographic and clinical data, including age, BMI, medical history, and other relevant clinical information, were collected. Due to the higher risks associated with multiple pregnancies compared to singleton pregnancies, 63 cases of multiple pregnancies were excluded after the follow-up of pregnancy outcomes. Additionally, 8 participants who terminated their pregnancies for non-medical reasons were excluded, and 54 participants who were lost to follow-up were removed from the analysis, leaving a final sample size of 475 participants. This study was approved by the Institutional Review Board (IRB) of the Shanghai Jiao Tong University School of Medicine, Renji Hospital Ethics Committee, under approval number [KY2019-056].

### Healthy dietary patterns

Women provided data on their preconception eating behaviors by completing the food frequency questionnaire (FFQ), which asked them to report the average frequency of consumption of each food item over the past year. Adherence to six *a priori*–defined dietary patterns was assessed using the following scores: (1) American Heart Association (AHA) dietary recommendations (4), (2) Trichopoulou Mediterranean diet (TMED) (12), (3) Panagiotakos Mediterranean diet (PMED) (13), (4) Alternate Mediterranean diet (AMED) (14), (5) the Healthy Eating Index 2015 (HEI-2015) (15), (6) Dietary Approaches to Stop Hypertension index (DASH) (16) (for detailed scoring, see Supplementary Table). Due to prior research suggesting that a plant-based diet (PBD) does not significantly impact pregnancy outcomes, it was excluded from the analysis (11).

In general, all six dietary patterns emphasize the consumption of whole grains, nuts, legumes, fruits, vegetables, fish, and olive oil or other monounsaturated fats, while discouraging the intake of red meat. The AHA, HEI-2015, and DASH indices also account for sodium and sugary drink consumption, both of which are discouraged. Some patterns further promote moderate alcohol intake and limit the consumption of saturated fats. Higher scores on these indices reflect greater adherence to the respective dietary patterns. Additionally, daily energy, protein, carbohydrate, and fat intake were estimated using the Chinese Food Composition Table, developed by the Institute of Nutrition and Food Safety, Chinese Center for Disease Control and Prevention (17).

### Outcome assessment

The primary outcome of this study was pregnancy loss, defined as a *β*-hCG level greater than 7 mIU/mL that did not result in a live birth, thus encompassing both biochemical and clinical pregnancy losses. Secondary outcomes included adverse pregnancy outcomes, defined as any conditions or complications during pregnancy or childbirth that negatively impact the health of the mother, fetus, or newborn. For this study, adverse pregnancy outcomes were categorized as follows: (1) Gestational diabetes mellitus (GDM): fasting blood glucose ≥5.1 mmol/L, or oral glucose tolerance test (OGTT) results meeting any of the following criteria: fasting ≥5.1 mmol/L, 1 h after glucose intake ≥10.0 mmol/L, or 2 h after glucose intake ≥8.5 mmol/L; (2) Hypertensive disorders of pregnancy (HDP): including gestational hypertension, unspecified hypertension, pre-eclampsia, and eclampsia; (3) Other adverse pregnancy outcomes: preterm birth, low birth weight, large for gestational age, fetal growth restriction (FGR), small for gestational age (SGA), and preterm premature rupture of membranes (PPROM). This study was reported in accordance with the Strengthening the Reporting of Observational Studies in Epidemiology (STROBE) guidelines for cohort studies (see Supplementary Table S1 for checklist).

### Statistical analysis

The study reported the baseline characteristics of the participants, and the distribution of these characteristics was compared across quartiles of dietary pattern scores and pregnancy outcomes. Covariates included age (continuous), preconception BMI (continuous), education level (college or above vs. below college), number of previous pregnancy losses (2, 3, or ≥4), moderate-to-vigorous physical activity (MVPA, minutes/week, continuous), smoking history (ever vs. never smoker), and total daily energy intake (kcal/day, continuous). Categorical variables were compared using the chi-square test, while continuous variables were assessed using analysis of variance (ANOVA). The relative risks of pregnancy loss and adverse pregnancy outcomes were estimated using log-binomial regression models, stratified by quartiles of dietary pattern scores. A significant association was defined as a p-trend value < 0.05. The regression models were adjusted for potential confounders, including age, preconception BMI, education level (less than college education, college education or higher), number of previous pregnancy losses, moderate to vigorous physical activity, smoking history, and total daily energy intake (kilocalories/day). P for trend was calculated by assigning the median value of each quartile of dietary score and modeling this variable as a continuous term in the multivariable log-binomial regression model. All statistical analyses were conducted using SPSS version 26.

## Results

Of the 475 participants enrolled in the study, 381 achieved a successful pregnancy, while 94 (19.8%) experienced pregnancy loss. The mean age of the participants was 30 years (standard deviation [SD] = 3 years), and the mean BMI was 21.41 kg/m^2^ (SD = 1.75 kg/m^2^). All participants were of Asian descent, with only 13 (2.7%) reporting a history of smoking. The majority of participants held a college degree (427 [89.9%]). Most women had experienced two miscarriages (414 [87.2%]), while 61 participants had three or more miscarriages.

Firstly, participants were categorized into two groups based on their pregnancy outcome: live birth or pregnancy loss. Baseline characteristics, including age, BMI, physical activity levels, and daily intake of total energy, protein, carbohydrates, and fats, were compared between the two groups (Table 1). We found that age, BMI, smoking history, educational level, and the number of previous pregnancy losses were not significantly associated with pregnancy loss. In contrast, the live birth group had significantly lower mean daily energy and fat intake, and higher levels of moderate-to-vigorous physical activity compared to the pregnancy loss group.

**Table 1:** Baseline characteristics of study participants with pregnancy loss.

Variables	Live birth	Pregnancy loss	*p* value
	(*N* = 381)	(*N* = 94)	
Age	30.29 ± 3.20	30.14 ± 3.55	0.704
Preconception BMI	21.33 ± 1.74	21.72 ± 1.76	0.057
Ever smoker	10 (2.60%)	3 (3.2%)	0.763
College education or higher	338 (88.70%)	89 (94.70%)	0.086
Number of previous pregnancy losses (*n*):			0.601
2	335 (87.9%)	79 (84.0%)	
3	37 (9.7%)	12 (12.8%)	
≥4	9 (75.0%)	3 (3.2%)	
Moderate to vigorous physical activity, min/wk	64.65 ± 34.20	56.81 ± 30.31	**0.043**
Energy intake, kcal/d	1631.71 ± 537.79	1779.65 ± 584.41	**0.027**
Protein intake, g/d	59.20 ± 22.81	64.29 ± 26.46	0.089
Carbohydrate intake, g/d	211.58 ± 80.77	214.63 ± 83.03	0.749
Fats intake, g/d	60.00 ± 29.15	69.86 ± 36.97	**0.006**

Values are presented as mean ± SD for continuous variables and *n* (%) for categorical variables. *p* values are from *t*-tests (continuous variables) or chi-square tests (categorical variables). Moderate-to-vigorous physical activity is measured in minutes/week (continuous). Smoking history is categorized as “ever smoker” (including current and former) vs. “never smoker.” Number of previous pregnancy losses is categorized as 2, 3, or ≥4.

Bold values indicate statistical significance (*p* < 0.05).

Subsequently, the relationship between dietary patterns and the risk of pregnancy loss was evaluated. Higher adherence to the AHA [adjusted relative risk (RR) (95% CI), highest quartile (Q4) vs. lowest quartile (Q1): 0.36 (0.17, 0.78), P-trend = 0.043], TMED [adjusted RR (95% CI), highest (Q4) vs. lowest quartile (Q1): 0.41 (0.19, 0.88), P-trend = 0.012], AMED [adjusted RR (95% CI), highest (Q4) vs. lowest quartile (Q1): 0.38 (0.17, 0.87), P-trend = 0.046], and HEI-2015 [adjusted RR (95% CI), highest (Q4) vs. lowest quartile (Q1): 0.45 (0.23, 0.86), P-trend = 0.008] dietary patterns were significantly associated with a reduced risk of pregnancy loss. In contrast, adherence to the PMED and DASH dietary patterns was not significantly related to pregnancy loss (Table 2).

**Table 2:** Relative risk (95% CI) of pregnancy loss by quartiles of AHA, TMED, PMED, AMED, HEI-2015, and DASH.

Dietary Pattern	Quartile (*n*, cases)	Adjusted RR (95% CI)	P for trend
AHA	Q1 (*n* = 120, cases = 28)	1.00 (reference)	
	Q2 (*n* = 118, cases = 29)	1.02 (0.55, 1.88)	
	Q3 (*n* = 120, cases = 26)	0.89 (0.47, 1.65)	
	Q4 (*n* = 117, cases = 11)	0.36 (0.17, 0.78)	0.043
TMED	Q1 (*n* = 140, cases = 34)	1.00 (reference)	
	Q2 (*n* = 137, cases = 34)	0.94 (0.53, 1.66)	
	Q3 (*n* = 113, cases = 14)	0.40 (0.20, 0.81)	
	Q4 (*n* = 85, cases = 12)	0.41 (0.19, 0.88)	0.012
PMED	Q1 (*n* = 119, cases = 27)	1.00 (reference)	
	Q2 (*n* = 119, cases = 25)	0.92 (0.49, 1.73)	
	Q3 (*n* = 119, cases = 16)	0.50 (0.25, 1.01)	
	Q4 (*n* = 118, cases = 26)	0.93 (0.49, 1.74)	0.221
AMED	Q1 (*n* = 171, cases = 41)	1.00 (reference)	
	Q2 (*n* = 131, cases = 27)	0.65 (0.36, 1.16)	
	Q3 (*n* = 103, cases = 16)	0.46 (0.23, 0.90)	
	Q4 (*n* = 70, cases = 10)	0.38 (0.17, 0.87)	0.046
HEI-2015	Q1 (*n* = 121, cases = 33)	1.00 (reference)	
	Q2 (*n* = 134, cases = 28)	0.62 (0.34, 1.12)	
	Q3 (*n* = 104, cases = 12)	0.30 (0.14, 0.63)	
	Q4 (*n* = 116, cases = 21)	0.45 (0.23, 0.86)	0.008
DASH	Q1 (*n* = 128, cases = 31)	1.00 (reference)	
	Q2 (*n* = 117, cases = 25)	0.82 (0.44, 1.52)	
	Q3 (*n* = 141, cases = 27)	0.70 (0.38, 1.29)	
	Q4 (*n* = 89, cases = 11)	0.38 (0.18, 0.84)	0.110

Adjusted for age (continuous, years), preconception BMI (continuous, kg/m^2^), education (college or above vs. below college), number of previous pregnancy losses (categorical: 2, 3, ≥4), moderate-to-vigorous physical activity (continuous, min/week), smoking history (ever vs. never smoker), and total energy intake (continuous, kcal/day).

Among the 381 participants who achieved a live birth, 42 women developed gestational diabetes mellitus (GDM), and 22 women experienced hypertensive disorders of pregnancy. Of these 22 HDP cases, 14 were gestational hypertension, 8 were pre-eclampsia and no cases of eclampsia were reported. Additionally, there were 6 cases of low birth weight, 1 case of placental abruption, 3 cases of fetal growth restriction, and 15 cases of preterm birth. These conditions were collectively classified as other adverse pregnancy outcomes. No significant differences in baseline characteristics were observed between participants with and without gestational diabetes, hypertensive disorders of pregnancy, or other adverse pregnancy outcomes (Table 3).

**Table 3:** Baseline characteristics of study participants with adverse pregnancy outcomes.

Live birth (*N* = 381)	Gestational diabetes mellitus (GDM)			Hypertensive disorders of pregnancy (HDP)			Other adverse pregnancy outcome (APO)		
Variables	Non-GDM	GDM	*p* value	Non-HDP	HDP	*p* value	Non-other APO	Other APO	*p* value
	(*N* = 339)	(*N* = 42)		(*N* = 359)	(*N* = 22)		(*N* = 356)	(*N* = 25)	
Age	30.29 ± 3.23	30.26 ± 3.01	0.947	30.22 ± 3.15	31.41 ± 3.88	0.173	30.26 ± 3.13	30.75 ± 4.18	0.579
Preconception BMI	21.30 ± 1.71	21.58 ± 1.99	0.384	21.35 ± 1.77	21.01 ± 1.30	0.373	21.31 ± 1.74	21.69 ± 1.76	0.313
Ever smoker	10 (2.9%)	0 (0.0%)	0.259	10 (2.8%)	0 (0.0%)	0.428	9 (2.5%)	1 (6.6%)	0.656
College education or higher	301 (88.8%)	37 (88.1%)	0.893	319 (88.9%)	19 (86.4%)	0.720	314 (88.2%)	24 (96.0%)	0.234
Number of previous pregnancy losses (*n*):			0.999			0.236			0.413
2	298 (87.9%)	37 (88.1%)		314 (87.5%)	21 (95.5%)		314 (88.2%)	21 (84%)	
3	33 (9.7%)	4 (10.8%)		37 (10.3%)	0 (0%)		33 (9.3%)	4 (16.0%)	
≥4	8 (2.4%)	1 (2.4%)		8 (2.2%)	1 (4.5%)		9 (2.5%)	0 (0.0%)	
Moderate to vigorous physical activity, min/wk	65.40 ± 34.46	58.57 ± 31.74	0.198	64.76 ± 34.00	62.73 ± 38.07	0.809	64.12 ± 19.02	72.5- ± 36.39	0.283
Energy intake, kcal/d	1628.02 ± 537.29	1661.43 ± 546.44	0.710	1635.70 ± 547.25	1566.53 ± 350.91	0.396	1630.26 ± 539.19	1652.17 ± 527.16	0.839
Protein intake, g/d	59.70 ± 23.12	55.14 ± 19.91	0.176	59.28 ± 22.92	57.76 ± 21.25	0.748	59.62 ± 23.00	52.75 ± 19.02	0.102
Carbohydrate intake, g/d	211.16 ± 81.26	214.97 ± 77.58	0.766	212.11 ± 81.46	202.82 ± 69.66	0.553	213.03 ± 81.52	189.93 ± 66.34	0.115
Fats intake, g/d	59.66 ± 29.07	62.71 ± 29.98	0.536	60.22 ± 29.93	56.27 ± 9.17	0.537	59.04 ± 28.48	74.25 ± 35.41	0.050

Values are presented as mean ± SD for continuous variables and *n* (%) for categorical variables. *p* values are from *t*-tests (continuous variables) or chi-square tests (categorical variables).

For gestational diabetes mellitus (GDM), higher adherence to the AHA [adjusted relative risk (RR) (95% CI), highest quartile (Q4) vs. lowest quartile (Q1): 0.28 (0.10, 0.75), P-trend = 0.006], TMED [adjusted RR (95% CI), highest (Q4) vs. lowest quartile (Q1): 0.12 (0.03, 0.56), P-trend = 0.049], AMED [adjusted RR (95% CI), highest (Q4) vs. lowest quartile (Q1): 0.15 (0.03, 0.71), P-trend = 0.005], and HEI-2015 [adjusted RR (95% CI), highest (Q4) vs. lowest quartile (Q1): 0.21 (0.08, 0.54), P-trend = 0.000] dietary patterns were significantly associated with a reduced risk of GDM (Table 4).

**Table 4:** Relative risk (95% CI) of GDM, hypertensive disorders of pregnancy, and other adverse pregnancy outcomes by quartiles of AHA, TMED, PMED, AMED, HEI-2015, and DASH.

Dietary pattern	Quartile	Gestational diabetes mellitus (*N* = 42/381)	Hypertensive disorders of pregnancy (*N* = 22/381)	Other adverse pregnancy outcomes (*N* = 25/381)
AHA	Q1	1	1	1
	Q2	1.03 (0.46,2.27)	0.59 (0.21,1.68)	0.42 (0.16,1.11)
	Q3	0.26 (0.09,0.75)	0.12 (0.02,0.57)	0.11 (0.02,0.51)
	Q3	0.28 (0.10,0.75)	0.12 (0.03,0.57)	0.04 (0.01,0.35)
	P trend	**0.043**	**0.008**	**0.001**
TMED	Q1	1	1	1
	Q2	0.57 (0.25,1.30)	0.22 (0.06,0.79)	0.46 (0.16,1.33)
	Q3	0.60 (0.26,1.39)	0.29 (0.09,0.95)	0.36 (0.11,1.13)
	Q3	0.12 (0.03,0.56)	0.09 (0.01,0.77)	0.33 (0.08,1.30)
	P trend	**0.049**	**0.014**	0.201
PMED	Q1	1	1	1
	Q2	0.73 (0.32,1.66)	0.44 (0.14,1.39)	0.78 (0.27,2.24)
	Q3	0.41 (0.16,1.01)	0.34 (0.10,1.16)	0.52 (0.17,1.56)
	Q3	0.35 (0.13,0.94)	0.27 (0.07,1.03)	0.27 (0.07,1.08)
	P trend	0.099	0.138	0.273
AMED	Q1	1	1	1
	Q2	1.02 (0.49,2.14)	0.21 (0.06,0.76)	0.44 (0.15,1.28)
	Q3	0.18 (0.05,0.65)	0.16 (0.03,0.74)	0.43 (0.14,1.33)
	Q3	0.15 (0.03,0.71)	0.10 (0.01,0.86)	0.12 (0.01,0.99)
	P trend	**0.005**	**0.008**	0.124
HEI-2015	Q1	1	1	1
	Q2	0.33 (0.15,0.75)	0.34 (0.11,1.04)	0.43 (0.15,1.27)
	Q3	0.06 (0.01,0.28)	0.35 (0.11,1.11)	0.45 (0.14,1.40)
	Q3	0.21 (0.08,0.54)	0.07 (0.01,0.58)	0.32 (0.09,1.12)
	P trend	**0.000**	**0.029**	0.223
DASH	Q1	1	1	1
	Q2	0.74 (0.32,1.75)	0.57 (0.20,1.61)	0.31 (0.09,1.04)
	Q3	0.64 (0.28,1.48)	0.20 (0.05,0.74)	0.37 (0.12,1.08)
	Q3	0.30 (0.10,0.98)	0.11 (0.01,0.85)	0.33 (0.09,1.14)
	P trend	0.246	0.032	0.111

Log-binomial regression models adjusted for: Age (continuous, years); Preconception BMI (continuous, kg/m^2^); Smoking history (ever vs. never); Education (college or above vs. below college); Number of previous pregnancy losses (categorical: 2, 3, ≥4); Moderate-to-vigorous physical activity (continuous, min/week); Total energy intake (continuous, kcal/day).

Bold values indicate statistical significance (*p* < 0.05).

A similar pattern was observed for hypertensive disorders of pregnancy (HDP), where higher adherence to the AHA [adjusted RR (95% CI), highest quartile (Q4) vs. lowest quartile (Q1): 0.12 (0.03, 0.57), P-trend = 0.008], TMED [adjusted RR (95% CI), highest (Q4) vs. lowest quartile (Q1): 0.09 (0.01, 0.77), P-trend = 0.014], AMED [adjusted RR (95% CI), highest (Q4) vs. lowest quartile (Q1): 0.10 (0.01, 0.86), P-trend = 0.008], and HEI-2015 [adjusted RR (95% CI), highest quartile (Q4) vs. lowest quartile (Q1): 0.07 (0.01, 0.58), P-trend = 0.029] dietary patterns were significantly associated with a lower risk of HDP (Table 4).

For other adverse pregnancy outcomes, only higher adherence to the AHA dietary pattern [adjusted relative risk (RR) (95% CI), highest quartile (Q4) vs. lowest quartile (Q1): 0.04 (0.01, 0.35), P-trend = 0.001] was significantly associated with a reduced risk of these outcomes. No significant association was observed between adherence to the TMED, PMED, AMED, HEI-2015, or DASH dietary patterns and the likelihood of other adverse pregnancy outcomes (Table 4).

## Discussion

This study provides a comparative analysis of the AHA diet and other dietary patterns in managing recurrent pregnancy loss (RPL). Our results indicate that higher adherence to the AHA dietary pattern is associated with a reduced risk of pregnancy loss, gestational diabetes mellitus (GDM), hypertensive disorders of pregnancy (HDP), and other adverse pregnancy outcomes. These findings suggest that, compared to other dietary patterns (TMED, AMED, HEI-2015, PMED, and DASH), the AHA diet may offer a protective effect by increasing the live birth rate and reducing pregnancy complications in women with recurrent pregnancy loss. We also find that lower levels of moderate-to-vigorous physical activity, higher daily intake of energy and fats during the preconception period was associated with an increased risk of pregnancy loss.

The role of dietary habits in reproductive health has gained increasing attention in recent years, with numerous studies suggesting that couples’ nutritional patterns can influence fertility and pregnancy outcomes (18, 19). Our findings are consistent with results from interventional studies evaluating the effect of dietary patterns during pregnancy. Notably, the IMPACT BCN randomized controlled trial demonstrated that a Mediterranean diet intervention in pregnant women significantly reduced the risk of small-for-gestational-age (SGA) newborns and other adverse perinatal outcomes. These findings reinforce the potential benefits of a high-quality, plant-based dietary pattern in supporting maternal-fetal health and align with our observational results. The stronger associations observed with the AHA dietary pattern may be partially attributable to its more rigorous scoring thresholds. Unlike other indices such as DASH, which emphasize general dietary balance, the AHA score assigns higher weight to stringent control of sodium intake (<1,500 mg/day), limited saturated fat (<6% of total energy), and strict avoidance of processed meats. These components may be particularly relevant in the context of metabolic and inflammatory pathways implicated in pregnancy loss and complications. While these scoring distinctions offer a plausible explanation, future studies are warranted to directly compare dietary indices and elucidate whether specific components—or overall restrictiveness—drive their relative effectiveness. Our findings contribute to this growing body of evidence by highlighting the potential benefits of dietary modifications, specifically the AHA diet, in managing recurrent pregnancy loss (20).

One key mechanism through which the AHA diet may impact RPL is its ability to improve metabolic disorders (21). Women with RPL are at an increased risk of metabolic disorders (22), including insulin resistance (23, 24) and polycystic ovary syndrome (PCOS) (25, 26), both of which are associated with a higher risk of miscarriage. We did not collect baseline cardiometabolic biomarkers such as blood pressure, glucose, or lipid levels, which may mediate the relationship between diet and pregnancy outcomes. Future studies incorporating these measures are warranted to elucidate potential mechanistic pathways. The AHA diet, rich in fiber and healthy fats, has been shown to regulate blood glucose levels and improve insulin sensitivity, which may help reduce the risk of RPL in women with these metabolic conditions. Additionally, the anti-inflammatory properties of the AHA diet could be beneficial in RPL. Chronic low-grade inflammation has been implicated in the pathophysiology of RPL (27, 28), and dietary patterns that reduce systemic inflammation may improve pregnancy outcomes (29). The AHA diet, which includes omega-3 fatty acids from sources like fish and nuts, has been shown to reduce inflammatory markers (30), suggesting that it may help to mitigate some of these risk factors, ultimately increasing the likelihood of a successful pregnancy. Our study lacked empirical biomarker data—such as inflammatory cytokines or insulin resistance indices—to directly support these mechanistic pathways. This represents a limitation, and future research incorporating relevant biomarkers will be essential to validate these proposed mechanisms.

In addition, previous studies have suggested that a high intake of vegetables, fruits, seafood, dairy products, eggs, and grains is associated with a reduced risk of miscarriage or improved pregnancy outcomes (18). However, the relationship between the consumption of red meat, fats and oils, and sugar substitutes, and the risk of miscarriage remains unclear (31–33). In our study, we found that higher daily intakes of energy and fats were associated with an increased risk of pregnancy loss. However, we did not specifically analyze the effects of individual food items on pregnancy outcomes.

Our study has several limitations that should be considered when interpreting the findings. First, as a single-center, observational cohort study, the ability to infer causality is inherently limited. Although we adjusted for multiple potential confounders, residual confounding due to unmeasured variables—such as socioeconomic status, physical activity, psychosocial stress, and cultural dietary norms—may still bias the observed associations. The lack of sensitivity analyses and power calculations also limits our ability to assess the robustness of the results. Future research incorporating prospective validation, causal inference methods, and sensitivity testing is warranted. Second, dietary intake was assessed using a semi-quantitative, self-administered FFQ, which is subject to recall bias, measurement error, and social desirability bias. Although the instrument has been validated in Chinese populations, inaccuracies in portion estimation and frequency reporting may have led to misclassification of dietary exposures. Additionally, dietary data were collected at a single time point before pregnancy, without accounting for potential changes throughout early pregnancy or during assisted conception treatments. This cross-sectional snapshot may not fully reflect the dynamic nature of diet and its evolving impact on pregnancy outcomes. Third, the exclusion of women with BMI < 18.5 or >30 kg/m^2^ and pre-existing chronic conditions may reduce heterogeneity but limits the applicability of our findings to the broader population, including those with higher metabolic risk. The cohort was relatively homogeneous—predominantly Asian and highly educated—further limiting generalizability to ethnically and socioeconomically diverse populations. Fourth, the dietary indices used in this study are based on distinct scoring systems, some relying on absolute thresholds (e.g., AHA, HEI-2015) and others on cohort-specific median intakes (e.g., TMED, AMED, PMED). This scoring heterogeneity may affect the direct comparability of the associations across patterns and should be interpreted with caution. Finally, although the TMED and AMED scores include alcohol consumption as a component, alcohol intake was negligible in our cohort and did not meaningfully contribute to score variability or outcomes. These limitations highlight the need for future studies with more diverse populations, repeated dietary assessments, objective biomarkers, and more comprehensive data on socioeconomic context to strengthen the understanding of how dietary patterns influence pregnancy outcomes.

## Conclusion

In this study, greater adherence to the AHA diet during the preconception period was associated with lower risks of pregnancy loss, gestational diabetes mellitus (GDM), hypertensive disorders of pregnancy (HDP), and other adverse pregnancy outcomes. In addition to the AHA pattern, higher adherence to the HEI-2015, AMED, and TMED dietary patterns was also linked to reduced risks of pregnancy loss, GDM, and HDP, while the PMED and DASH patterns showed no significant associations with these outcomes. Furthermore, higher levels of moderate-to-vigorous physical activity, along with reduced daily energy and fat intake, were associated with a higher likelihood of live birth. These findings provide valuable insights into the potential role of healthy dietary patterns in improving pregnancy outcomes and underscore the need for further research to explore how these patterns may impact female fertility.

## Data Availability

The raw data supporting the conclusions of this article will be made available by the authors, without undue reservation.
