# Supplementary material for: Dietary patterns and recurrent pregnancy loss: a comparison of the American Heart Association diet, Mediterranean diet and others
Source: Front Nutr. 2025 Jun 4;12:1565107. doi: 10.3389/fnut.2025.1565107 (PMC12173877; doi:10.3389/fnut.2025.1565107)
Supplement: Supplementary file 2 [file Table_2.docx]

# STROBE Checklist for Cohort Studies

| Item No. | Section/Topic | Item Description | Location in Manuscript |
| --- | --- | --- | --- |
| 1 | Title and abstract | Indicate the study’s design with a commonly used term in the title or the abstract. | Title; Abstract |
| 2 | Background/rationale | Explain the scientific background and rationale for the investigation being reported. | Introduction, Paragraphs 1–3 |
| 3 | Objectives | State specific objectives, including any prespecified hypotheses. | Introduction, Final Paragraph |
| 4 | Study design | Present key elements of study design early in the paper. | Methods, First Paragraph |
| 5 | Setting | Describe the setting, locations, and relevant dates, including periods of recruitment, exposure, follow-up, and data collection. | Methods, 'Study Population' |
| 6a | Participants | Give the eligibility criteria, and the sources and methods of selection of participants. Describe methods of follow-up. | Methods, Inclusion/Exclusion Criteria |
| 7 | Variables | Clearly define all outcomes, exposures, predictors, potential confounders, and effect modifiers. | Methods, 'Dietary Assessment' and 'Outcome Measures' |
| 8 | Data sources/measurement | For each variable of interest, give sources of data and details of methods of assessment. | Methods, 'Dietary Assessment' and 'Outcome Measures' |
| 9 | Bias | Describe any efforts to address potential sources of bias. | Methods, 'Statistical Analysis'; Discussion, Limitations |
| 10 | Study size | Explain how the study size was arrived at. | Methods, 'Sample Size Calculation' |
| 11 | Quantitative variables | Explain how quantitative variables were handled in the analyses. | Methods, 'Statistical Analysis' |
| 12a | Statistical methods | Describe all statistical methods, including those used to control for confounding. | Methods, 'Statistical Analysis' |
| 12b |  | Describe any methods used to examine subgroups and interactions. | Methods, 'Statistical Analysis' |
| 12c |  | Explain how missing data were addressed. | Methods, 'Statistical Analysis' |
| 12d |  | If applicable, explain how loss to follow-up was addressed. | Not applicable (no longitudinal follow-up) |
| 12e |  | Describe any sensitivity analyses. | Discussion, 'Limitations' |
| 13a | Participants | Report numbers of individuals at each stage of study. | Results, Flowchart Figure |
| 13b |  | Give reasons for non-participation at each stage. | Results, Flowchart Figure |
| 13c |  | Consider use of a flow diagram. | Figure 1 |
| 14a | Descriptive data | Give characteristics of study participants and potential confounders. | Results, Table 1 |
| 14b |  | Indicate number of participants with missing data for each variable of interest. | Results, Table 1 footnote |
| 14c |  | Summarize follow-up time. | Not applicable |
| 15 | Outcome data | Report numbers of outcome events or summary measures over time. | Results, Table 2–4 |
| 16a | Main results | Give unadjusted and adjusted estimates with precision and confounder info. | Results, Table 3–4 |
| 16b |  | Report category boundaries when continuous variables were categorized. | Methods, 'Statistical Analysis' |
| 16c |  | Translate estimates of relative risk into absolute risk if relevant. | Not applicable |
| 17 | Other analyses | Report other analyses done (e.g., subgroups, interactions, sensitivity). | Results, Subgroup Analyses; Supplementary Tables |
| 18 | Key results | Summarize key results with reference to study objectives. | Discussion, First Paragraph |
| 19 | Limitations | Discuss study limitations and bias or imprecision. | Discussion, 'Limitations' Section |
| 20 | Interpretation | Provide cautious overall interpretation of results. | Discussion, Final Paragraphs |
| 21 | Generalisability | Discuss generalisability (external validity). | Discussion, 'Limitations' Section |
| 22 | Funding | Give funding sources and roles. | Funding Statement |
